# Intracellular zinc protects Kv7 K^+^ channels from Ca^2+^/calmodulin-mediated inhibition

**DOI:** 10.1016/j.jbc.2022.102819

**Published:** 2022-12-20

**Authors:** Xinhe Yang, Shuai Chen, Shuo Zhang, Sai Shi, Rui Zong, Yiting Gao, Bingcai Guan, Nikita Gamper, Haixia Gao

**Affiliations:** 1 Department of Pharmacology, Center for Innovative Drug Research and Evaluation, Institute of Medical Science and Health, The Hebei Collaboration Innovation Center for Mechanism, Diagnosis and Treatment of Neurological and Psychiatric Disease, The Key Laboratory of Neural and Vascular Biology, Ministry of Education, Hebei Medical University, Shijiazhuang, Hebei, China; 2 CSPC ZhongQi Pharmaceutical Technology (Shijiazhuang) Co, Ltd, Shijiazhuang, Hebei, China; 3 Tianjin Key Laboratory of Function and Application of Biological Macromolecular Structures, School of Life Sciences, Tianjin University, Tianjin, China; 4 Faculty of Biological Sciences, School of Biomedical Sciences, University of Leeds, Leeds, UK

**Keywords:** potassium channel, calcium, calmodulin, zinc, electrophysiology, AMPA, α-amino-3-hydroxy-5-methyl-4-isoxazolepropionic acid, CaM, calmodulin, cDNA, complementary DNA, CHO, Chinese hamster ovary cell, Co-IP, coimmunoprecipitation, DMEM, Dulbecco's modified Eagle's medium, GPCR, G protein–coupled receptor, IgG, immunoglobulin G, PIP_2_, phosphatidylinositol 4,5-bisphoshpate, PLC, phospholipase C, TPEN, *N*,*N*,*N*′,*N*′,tetrakis(2-pyridylmethyl)etylenediaminepentaethylene, Zn, zinc, ZnPy, zinc pyrithione

## Abstract

Zinc (Zn) is an essential trace element; it serves as a cofactor for a great number of enzymes, transcription factors, receptors, and other proteins. Zinc is also an important signaling molecule, which can be released from intracellular stores into the cytosol or extracellular space, for example, during synaptic transmission. Amongst cellular effects of zinc is activation of Kv7 (KCNQ, M-type) voltage-gated potassium channels. Here, we investigated relationships between Kv7 channel inhibition by Ca^2+^/calmodulin (CaM) and zinc-mediated potentiation. We show that Zn^2+^ ionophore, zinc pyrithione (ZnPy), can prevent or reverse Ca^2+^/CaM-mediated inhibition of Kv7.2. In the presence of both Ca^2+^ and Zn^2+^, the Kv7.2 channels lose most of their voltage dependence and lock in an open state. In addition, we demonstrate that mutations that interfere with CaM binding to Kv7.2 and Kv7.3 reduced channel membrane abundance and activity, but these mutants retained zinc sensitivity. Moreover, the relative efficacy of ZnPy to activate these mutants was generally greater, compared with the WT channels. Finally, we show that zinc sensitivity was retained in Kv7.2 channels assembled with mutant CaM with all four EF hands disabled, suggesting that it is unlikely to be mediated by CaM. Taken together, our findings indicate that zinc is a potent Kv7 stabilizer, which may protect these channels from physiological inhibitory effects of neurotransmitters and neuromodulators, protecting neurons from overactivity.

Zinc (Zn) is the second most abundant trace metal in the body (after iron) with functions ranging from intracellular messaging to regulation of protein structure and enzymatic activity (1). Plasma levels of zinc are low and account only for a small fraction of total body zinc, which is mostly stored intracellularly. In cells, zinc is bound not only to proteins such as metallothioneins, which can be released upon oxidation, but also to many other protein types (zinc finger proteins, many enzymes, etc.) (1). Zinc is transported to the cytoplasm from the extracellular space and from organelles *via* ZIP transporters (Zrt-, Irt-related Proteins) and is removed from the cytoplasm into the extracellular space or organelles *via* zinc transporter proteins (ZnT) (1). In the central nervous system, particularly in the hippocampus, zinc is strongly accumulated *via* ZnT3 into glutamatergic synaptic vesicles, resulting in a luminal concentration exceeding 1 mM (2). Zn^2+^ released into the synaptic cleft during synaptic transmission can rapidly enter postsynaptic neurons *via*, for example, Ca^2+^-permeable α-amino-3-hydroxy-5-methyl-4-isoxazolepropionic acid (AMPA) receptors (3, 4, 5) and some other cationic channels, such as TRPM7 (transient receptor potential cation channel subfamily M member 7) (6). The processes of synaptic release and reuptake of Zn^2+^ have important physiological implications for hippocampal information processing (5, 7, 8). An excessive cytosolic zinc accumulation during pathological overactivity was suggested to contribute to neuronal death, for example, during brain ischemia (3, 4, 5).

One of the mechanisms controlling neuronal excitability, especially during periods of overactivity, is M-type (KCNQ, Kv7) K^+^ channels (9). The channels give rise to noninactivating K^+^ currents with slow kinetics and a very negative activation threshold (negative to −60 mV). These features allow Kv7 channels to remain partially active at voltages near the resting membrane potential of a neuron and strongly influence excitability (10, 11). Kv7 channel inhibition leads to increased excitability, whereas long-term losses of KCNQ channel expression or activity often result in debilitating excitability disorders, such as epilepsy, deafness, pain, or arrhythmias (9, 10, 12). Conversely, M channel enhancers reduce excitability and were clinically used as anticonvulsants (retigabine) or analgesics (flupirtine), although these drugs are now discontinued because of side effects (13).

Kv7 channels are subject to multifaceted regulation and modulation by an array of neurotransmitters, neuromodulators, and other physiologically active compounds (reviewed in Ref. (9)). One prominent pathway for such modulation is mediated by G protein–coupled receptors (GPCRs) acting *via* G_q/11_-phospholipase C (PLC) signaling cascades. For their activity, Kv7 channels require a cofactor, membrane phosphoinositide, phosphatidylinositol 4,5-bisphoshpate (PIP_2_) (14, 15). Accordingly, G_q/11_PCR-mediated PIP_2_ depletion is one of the major mechanisms of excitation by endogenous neurotransmitters and neuromodulators, such as acetylcholine, histamine, glutamate, etc. (9, 10, 11). In addition to direct PIP_2_ depletion, Kv7 activity can be controlled by PKC-induced phosphorylation (16) or Ca^2+^/calmodulin (CaM)-mediated modulation (17, 18, 19), which, in turn, may act by modulating channel PIP_2_ affinity (reviewed in Ref. (9)).

Interestingly, Kv7 channel activity is strongly potentiated by intracellular Zn^2+^ (20) and zinc ionophores (21). Zinc dramatically reduces Kv7 channel PIP_2_ dependence, allowing normal channel activity in severely PIP_2_-depleted membranes (20). It is yet to be established whether in the presence of Zn^2+^, Kv7 channels become truly PIP_2_ insensitive or their PIP_2_ affinity becomes too high to respond to physiological fluctuations in PIP_2_ levels, but even strong PIP_2_ depletion with *Ciona intestinalis* voltage sensor–containing phosphatase or PLC is not sufficient to inhibit Kv7 channels in the presence of sufficient cytosolic levels of free Zn^2+^ (20). Intracellular Zn^2+^ or zinc ionophores also significantly reduced GPCR-mediated Kv7 channel inhibition (20, 22, 23). In this study, we investigated the relationships between zinc-mediated Kv7 channel potentiation and inhibition by Ca^2+^/CaM. We reasoned that since increased synaptic activity (at least in hippocampus) is associated with intracellular Zn^2+^ accumulation and also with Ca^2+^ influx through ionotropic receptors, such as AMPA, stabilization of Kv7 channel activity by zinc may serve a protective function preventing hyperexcitability.

## Results

### Zinc ionophore prevents Kv7.2 inhibition by intracellular Ca^2+^

**Figure 1:**
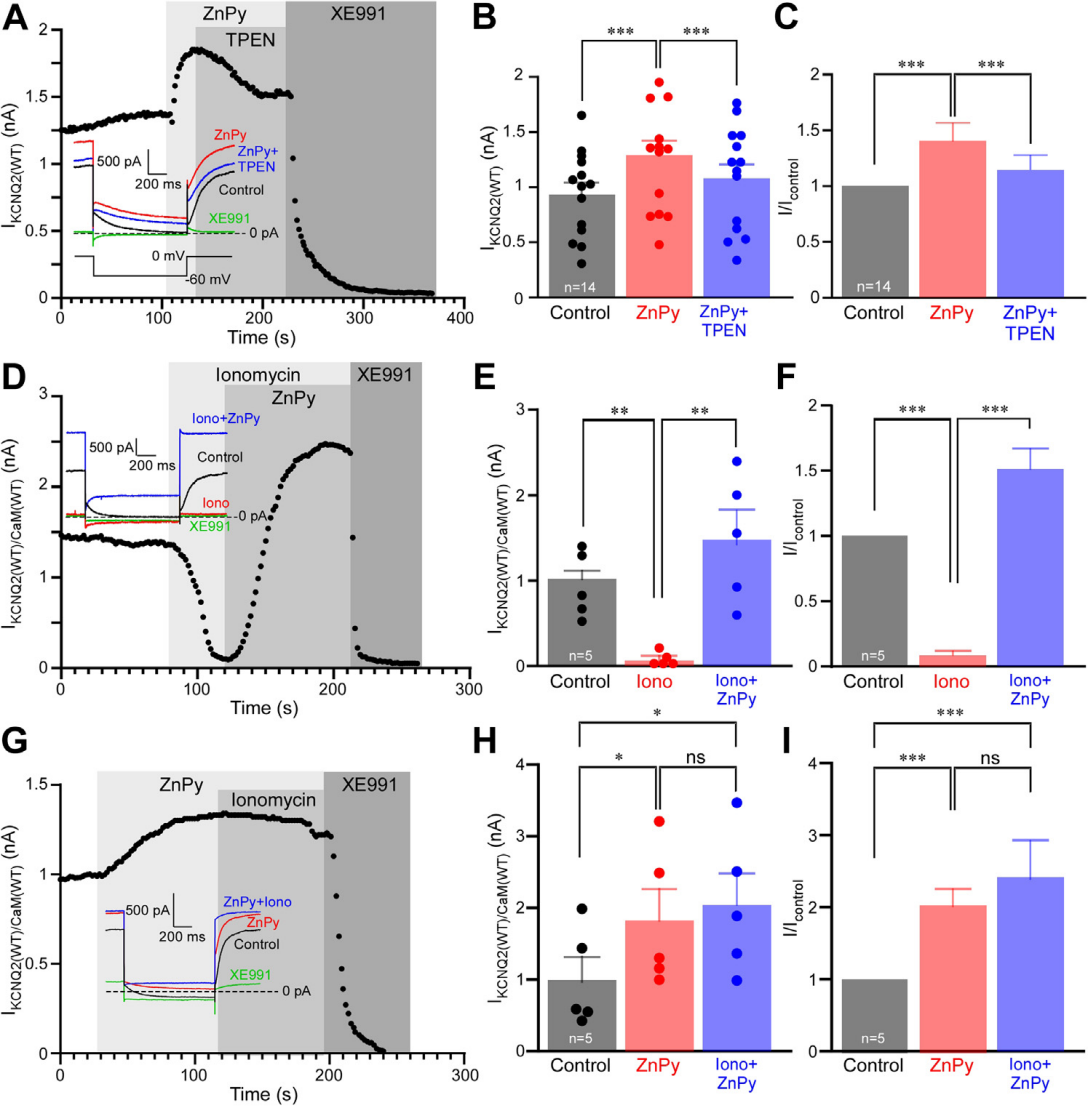
**Intracellular zinc (Zn) delivery abolishes Ca**^**2+**^**-induced inhibition of Kv7.2.***A*, perforated patch-clamp recording from Kv7.2-transfected Chinese hamster ovary (CHO) cell showing the time course for the effects of Zn^2+^ ionophore, zinc pyrithione (ZnPy) (10 μM), and Zn^2+^ chelator, TPEN (20 μM), as labeled. At the end of the recording, a specific Kv7 channel inhibitor, XE991 (10 μM), was applied. Examples of current traces are shown in the *inset* with the voltage protocol shown underneath. *Vertical gray bars* indicate periods of drug application. *B* and *C*, summary of the experiments shown in *A*. Mean current amplitudes are summarized in *B*, and normalized current amplitudes (relative to basal amplitude, *I*_control_) are summarized in *C*; n = 14. *Asterisks* depict a significant difference between the groups indicated by *connector lines*; ∗∗∗*p* < 0.001 (repeated-measures ANOVA with Bonferroni post hoc test). *D*, CHO cells were cotransfected with Kv7.2 and calmodulin (CaM); time course of the effects of Ca^2+^ ionophore, ionomycin (5 μM), ZnPy (10 μM), and XE991 (10 μM) is shown. Recording conditions and labeling are similar to that used in *A*. *E* and *F*, summary of experiments shown in *D*, n = 5. *Asterisks* depict a significant difference between the groups indicated by *connector lines*; ∗*p* < 0.05, ∗∗∗*p* < 0.001 (repeated-measures ANOVA with Bonferroni post hoc test). *G*, experiment similar to that shown in *D*, but ZnPy was applied first, followed by the application of ionomycin (still in the presence of ZnPy). *H* and *I*, summary of experiments shown in *G*, n = 5. *Asterisks* depict a significant difference between the groups indicated by *connector lines*; ∗*p* < 0.05, ∗∗∗*p* < 0.001 (repeated-measures ANOVA with Bonferroni post hoc test). TPEN, *N*,*N*,*N*′,*N*′,tetrakis(2-pyridylmethyl)ethylenediaminepentaethylene.

In our recent study, we demonstrated that Zn^2+^ ionophores, such as zinc pyrithione (ZnPy), pyrrolidinedithiocarbamate, diiodo-8-hydroxyquinoline, and some others, augment Kv7 channel currents by delivering free Zn^2+^ into the cytosol (20). Here, we used this experimental protocol to test how raising intracellular free Zn^2+^ affects inhibition of Kv7 channels by Ca^2+^. In perforated patch voltage clamp recordings from Chinese hamster ovary (CHO) cells transiently transfected with *KCNQ2* complementary DNA (cDNA), bath application of ZnPy (10 μM) augmented steady-state current amplitude at 0 mV by 1.41 ± 0.04-fold (Fig. 1, *A*–*C*; *p* < 0.001, n = 14). Zn^2+^ chelator *N*,*N*,*N*′,*N*′,tetrakis(2-pyridylmethyl)ethylenediaminepentaethylene (TPEN; 20 μM), applied in the presence of ZnPy, characteristically reversed the augmentation without inhibiting current amplitude below the basal level (Fig. 1, *A*–*C*). Although the reversal with 20 μM TPEN was almost complete, there was a degree of variability; at 30 μM TPEN completely reversed ZnPy augmentation (Fig. S1, *A*–*C*). The reversibility of ZnPy effect by Zn^2+^ chelator suggests that the active moiety mediating Kv7.2 augmentation is indeed Zn^2+^. We also tested lower concentrations of ZnPy (Fig. S1, *D–F*), and even at 500 nM, significant augmentation of Kv7.2 current was observed.

Next, we expanded this protocol to test the effect of Zn^2+^ elevation with ZnPy on Kv7 inhibition by Ca^2+^. Since CaM is required for Ca^2+^-dependent modulation of Kv7 channels (17, 18, 24, 25), we coexpressed Kv7.2 with CaM. Consistent with previous reports (17, 18, 26), application of Ca^2+^ ionophore, ionomycin (5 μM), in the presence of 2 mM extracellular Ca^2+^ produced sharp, nearly complete, inhibition of Kv7.2 current amplitude from 0.95 ± 0.17 nA to 0.09 ± 0.04 nA (Fig. 1, *D*–*F*; *p* < 0.001, n = 5). Application of ZnPy (still in the presence of Ca^2+^/ionomycin) induced rapid recovery of Ca^2+^/ionomycin-induced inhibition to the levels significantly higher than basal level (Fig. 1, *D*–*F*). Compared with basal level, current amplitude was increased by 1.53 ± 0.14-fold, which was comparable with the efficacy of ZnPy to augment Kv7.2 current amplitude in control conditions (Fig. 1, *A*–*C*). Thus, intracellular Zn^2+^ delivery was able to completely remove Ca^2+^/CaM-mediated inhibition. This was even more evident in the experiment where we applied ZnPy first, waited until Kv7.2 current amplitude increase reached a plateau, and then applied ionomycin (still in the presence of ZnPy). Under these conditions, ionomycin failed to produce any inhibition (Fig. 1, *G*–*I*). In our previous experiments, 5 μM ionomycin induced [Ca^2+^]_i_ transients in the range of 500 nM in CHO cells under our experimental conditions (18). Since such transients are variable and dynamic, we also measured responses to ZnPy in two steady-state conditions: we performed whole-cell patch-clamp experiments with intracellular solutions with cytosolic-free Ca^2+^ concentrations clamped by EDTA to 50 nM and 1 μM, to mimic “low” (subbasal) and “high” (near-maximal concentration during Ca^2+^ signaling event), physiological scenarios, respectively (Fig. S1, *G*–*J*). In both scenarios, ZnPy induced a robust Kv7.2 current augmentation; expectedly smaller currents recorded with 1 μM [Ca^2+^]_i_ were augmented to the level of basal currents recorded in 50 nM [Ca^2+^]_i_ (Fig. S1*I*).

**Figure 2:**
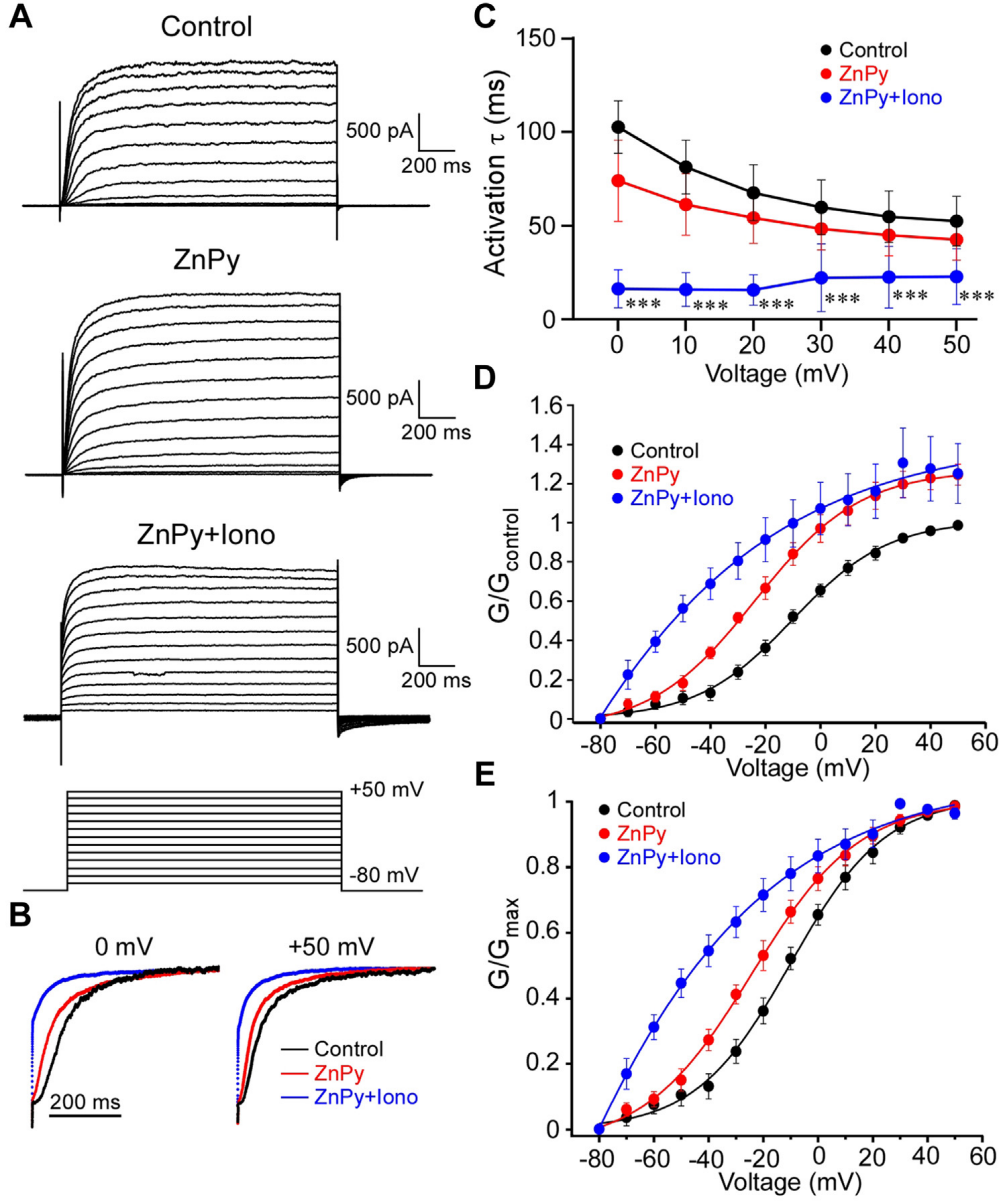
**In the presence of Zn**^**2+**^**and Ca**^**2+**^**ionophores, Kv7.2 is locked in the open state.***A*, Chinese hamster ovary (CHO) cells were cotransfected with Kv7.2 and calmodulin (CaM) and the effect of Zn^2+^ and Ca^2+^ ionophores (10 μM zinc pyrithione [ZnPy] and 5 μM ionomycin, respectively) on the channel voltage dependence, and kinetics were investigated. Shown are example current traces in control conditions (*top*), in the presence of ZnPy (*middle*), and in the presence of both ZnPy and ionomycin (*bottom*). Currents were elicited by the voltage protocol shown at the *bottom*. *B*, first 400 ms of the current traces elicited by voltage step from −80 mV to 0 mV (*left*) and +50 mV (*right*) under control, ZnPy and ZnPy + ionomycin conditions are superimposed and scaled to match in order to highlight changes in kinetics. *C*, summary of the effects of ZnPy and ionomycin on the activation time constant (τ) at different activation voltages; τs were obtained by fitting the traces with an exponential function. *Asterisks* depict a significant difference from control; ∗∗∗*p* < 0.001 (repeated-measures ANOVA with Bonferroni post hoc test). *D* and *E*, effects of ZnPy and ionomycin on the voltage dependence of Kv7.2. Conductance–voltage relationships normalized to *G*_control_ at +50 mV (*D*) or to *G*_max_ for each condition (*E*) are shown.

An interesting observation from the experiments reported in Figure 1 was that while after ZnPy treatment Kv7.2 current retained its slow activation and inactivation kinetics, when recordings were made in the simultaneous presence of both Zn^2+^ and Ca^2+^ ionophores, the currents lost these features almost entirely, allowing instantaneous change to voltage in either direction (*cf*. traces shown in *insets* in Fig. 1 [panels *A*, *D*, and *G*]). We investigated this further by comparing activation kinetics and current–voltage relationships of Kv7.2 currents in control conditions, in the presence of ZnPy or in the presence of both, ZnPy and ionomycin (Fig. 2). As noted, in the presence of both ionophores, a principal component of the Kv7.2 currents displayed instantaneous opening (Fig. 2, *A* and *B*). The remaining, slower-activating, fraction activated several-fold faster, as compared with control conditions, across the voltages tested (Fig. 2, *B* and *C*). Consistent with earlier reports (20, 21), ZnPy produced significant leftward shift of the Kv7.2 activation curve (from −10.93 mV to −22.66 mV). However, in the simultaneous presence of both ionophores, the current–voltage relationships underwent a further leftward shift and acquired significant linear component (Fig. 2, *D* and *E*; V_1/2_ = −43.05 mV). Both, dramatic acceleration of current kinetics and loss of voltage dependence, suggest that in the presence of both Ca^2+^ and Zn^2+^, the channel is being locked in the open state and no longer controlled by its voltage sensor. Of note, as evident from Figure 1, *D* and *G*, the current through these constitutively open channels was completely blocked by the selective Kv7 channel inhibitor, XE991 (10 μM), confirming that it was indeed generated by the Kv7.2.

We also tested if Ca^2+^-induced inhibition of a native neuronal M current can be rescued by Zn^2+^. In perforated-patch experiments, 5 μM ionomycin almost completely inhibited M-like outward XE991-sensitive current, and well over 50% of this inhibition was recovered by 10 μM ZnPy (Fig. S2).

### Reducing CaM binding to Kv7.2 does not compromise zinc-induced potentiation

**Figure 3:**
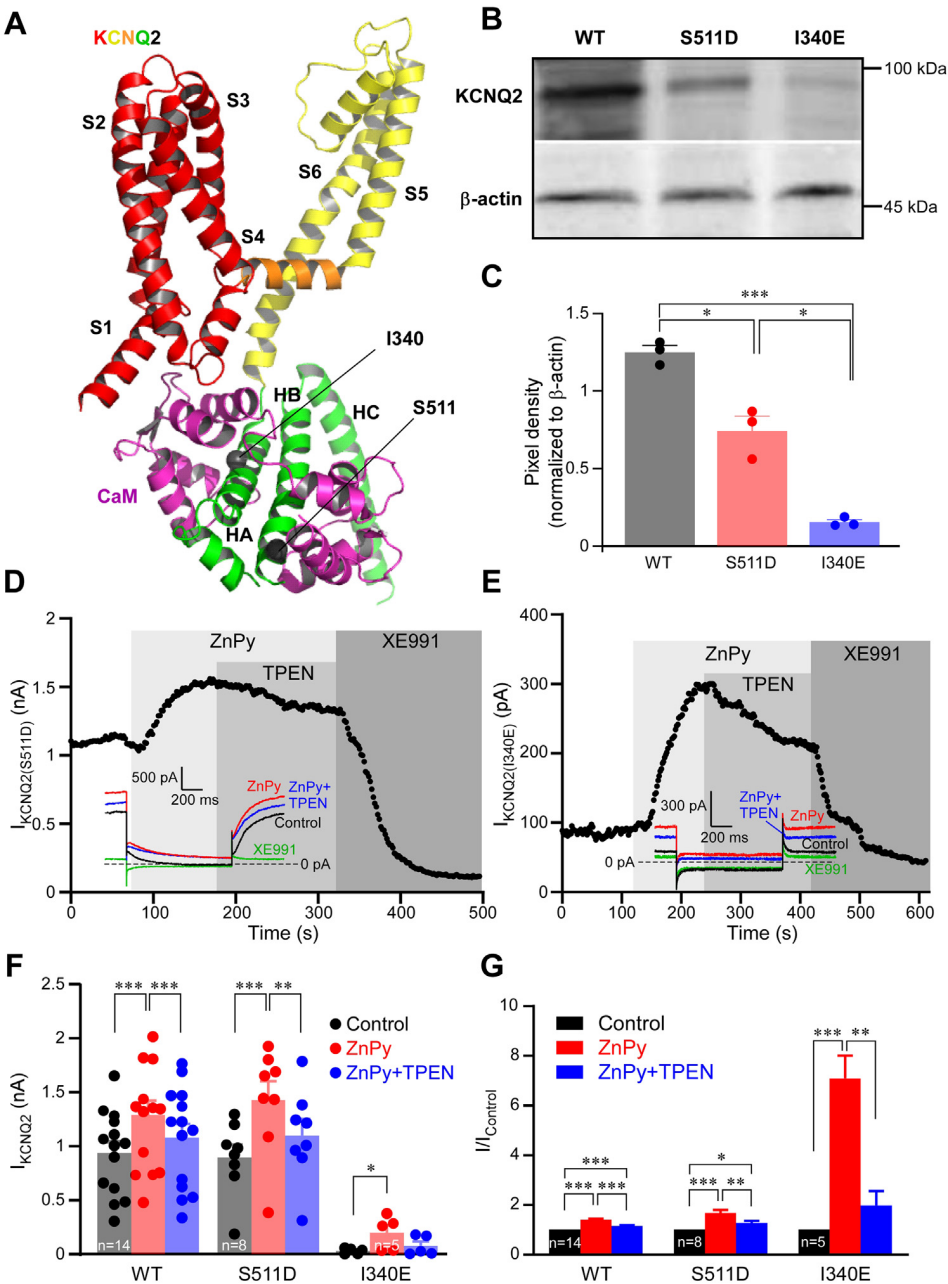
**Reducing calmodulin (CaM) binding to Kv7.2 does not prevent zinc-induced potentiation.***A*, structure of a single Kv7.2 subunit in complex with CaM (*purple*) with amino acid residues within helix A (I340) and helix B (S511), shown to be necessary for CaM binding (41, 42), indicated; structure is based on the human KCNQ2-CaM structure (Protein Data Bank ID: 7CR3). *B* and *C*, Chinese hamster ovary (CHO) cells were transfected with FLAG-tagged WT Kv7.2, Kv7.2 (I340E) or hemagglutinin-tagged Kv7.2 (S511D), and the membrane fraction of proteins was analyzed by Western blot. *B*, shows example experiment. *C*, displays mean band intensities, normalized to the housekeeping protein, β-actin. *Asterisks* depict a significant difference between the groups indicated by *connector lines*; ∗*p* < 0.05, ∗∗∗*p* < 0.001 (one-way ANOVA with Bonferroni post hoc test). *D* and *E*, perforated patch-clamp recordings from CHO cells transfected with either Kv7.2 (S511D) (*D*) or Kv7.2 (I340E) (*E*), showing the time courses for the effects of ZnPy (10 μM), TPEN (20 μM), and XE991 (10 μM), as indicated with *vertical gray bars*. Recording conditions and labeling are similar to that used for Figure 1*A*. *F* and *G*, summary of the experiments shown in *D* and *E*. Mean current amplitudes are summarized in *F*, and normalized current amplitudes (relative to *I*_control_) are summarized in *G*; number of experiments is indicated within the *bars*. *Asterisks* depict a significant difference between the groups indicated by *connector lines*; ∗*p* < 0.05, ∗∗*p* < 0.01, and ∗∗∗*p* < 0.001 (repeated-measures ANOVA with Bonferroni post hoc test). TPEN, *N*,*N*,*N*′,*N*′,tetrakis(2-pyridylmethyl)ethylenediaminepentaethylene.

We next thought to investigate if reducing CaM binding to the Kv7 channel would affect the modulation of Kv7 channels by Ca^2+^ and Zn^2+^. All Kv7 channels require CaM for functional assembly and trafficking (27, 28, 29, 30) as well as for modulation by Ca^2+^ (17, 25, 26, 28, 31, 32, 33). CaM binds simultaneously to A and B helixes of the Kv7 C terminus, which, when bound to CaM, adopts antiparallel coiled coil (Fig. 3*A*) (24, 34, 35, 36, 37, 38, 39, 40). It was shown previously that I340E substitution in helix A or S511D substitution in helix B (Fig. 3*A*) reduce CaM binding to Kv7.2 and also reduce plasma membrane expression of the channels as well as the whole cell currents generated (41, 42). We used these mutants to test if disturbed CaM binding affects the efficacy of zinc-mediated channel potentiation. When overexpressed in CHO cells, FLAG-tagged WT Kv7.2 and Kv7.2 (I340E) or hemagglutinin-tagged Kv7.2 (S511D) proteins were detectable by Western blot in the membrane fraction, although the expression of the mutants (especially I340E) was significantly reduced (Fig. 3, *B* and *C*), confirming reduced membrane trafficking of the mutants. Kv7.2 (S511D) generated sizable M-like current, which was potentiated by ZnPy to a similar extent (1.67 ± 0.13-fold, n = 8; Fig. 3, *D* and *G*), as compared with the WT Kv7.2 (1.41 ± 0.04-fold, n = 14; Fig. 1, *A*–*C*). The I340E mutant produced almost no current under basal conditions; however, when ZnPy was applied, there was a clear increase in the whole-cell current amplitude (0 mV), which was reversible by TPEN and sensitive to XE991 (Fig. 3, *E* and *G*). This was a surprising observation since there were clearly much fewer membrane-localized I340E channels (Fig. 3, *B* and *C*), and this mutant was early characterized as nonfunctional (41, 42).

**Figure 4:**
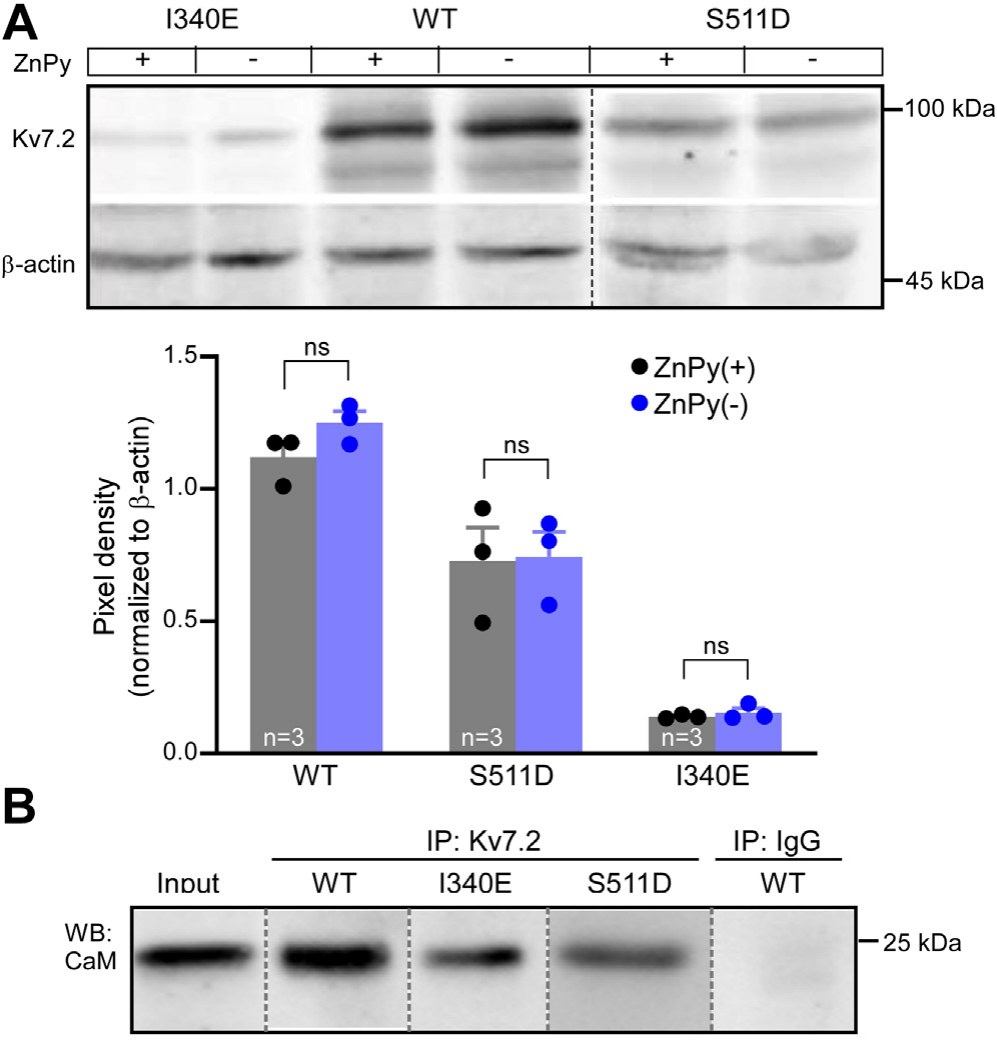
**Zinc does not acutely affect membrane abundance of Kv7.2.***A*, Western blot analysis of the membrane protein fraction of Chinese hamster ovary (CHO) cells transfected with FLAG-tagged WT Kv7.2 or Kv7.2 (I340E) or hemagglutinin -tagged Kv7.2 (S511D) and incubated for 2 min in the presence of 10 μM zinc pyrithione (ZnPy) or the vehicle (extracellular solution). *Bar graph* below shows mean band intensities, normalized to β-actin; ns (not significant) depicts *p* > 0.05 (paired *t* test) for groups indicated by *connector lines*. *B*, coimmunoprecipitation with calmodulin (CaM) of the FLAG-tagged WT Kv7.2 or Kv7.2 (I340E) or hemagglutinin-tagged Kv7.2 (S511D) using corresponding anti-FLAG or antihemagglutinin antibodies (see Experimental procedures section).

Importantly, membrane abundance of either Kv7.2 channels tested (WT, I340E, and S511D) was not affected by the ZnPy treatment (Fig. 4*A*). We also confirmed the effect of the mutations on Kv7.2–CaM interaction (Fig. 4*B*). In a coimmunoprecipitation (co-IP) experiment, FLAG-tagged WT Kv7.2 and Kv7.2 (I340E) or hemagglutinin-tagged Kv7.2 (S511D) were coexpressed with CaM and immunoprecipitated using the corresponding antibodies (anti-FLAG or antihemagglutinin, respectively); coprecipitated CaM was then detected using Western blot. As expected, both mutants bound CaM weaker (Fig. 4*B*), yet, CaM was still detectable. We interpret these results as that a fraction of the mutant channels does reach plasma membrane; these are likely to be still CaM bound. In the case of I340E, the number of functional channels at the plasma membrane is particularly low, and their basal activity (even at saturating voltages) is miniscule but can still be augmented by zinc. Interestingly, the relative efficacy of ZnPy to potentiate these I340E channels was significantly higher, as compared with WT Kv7.2 (Fig. 3, *E* and *G*).

**Figure 5:**
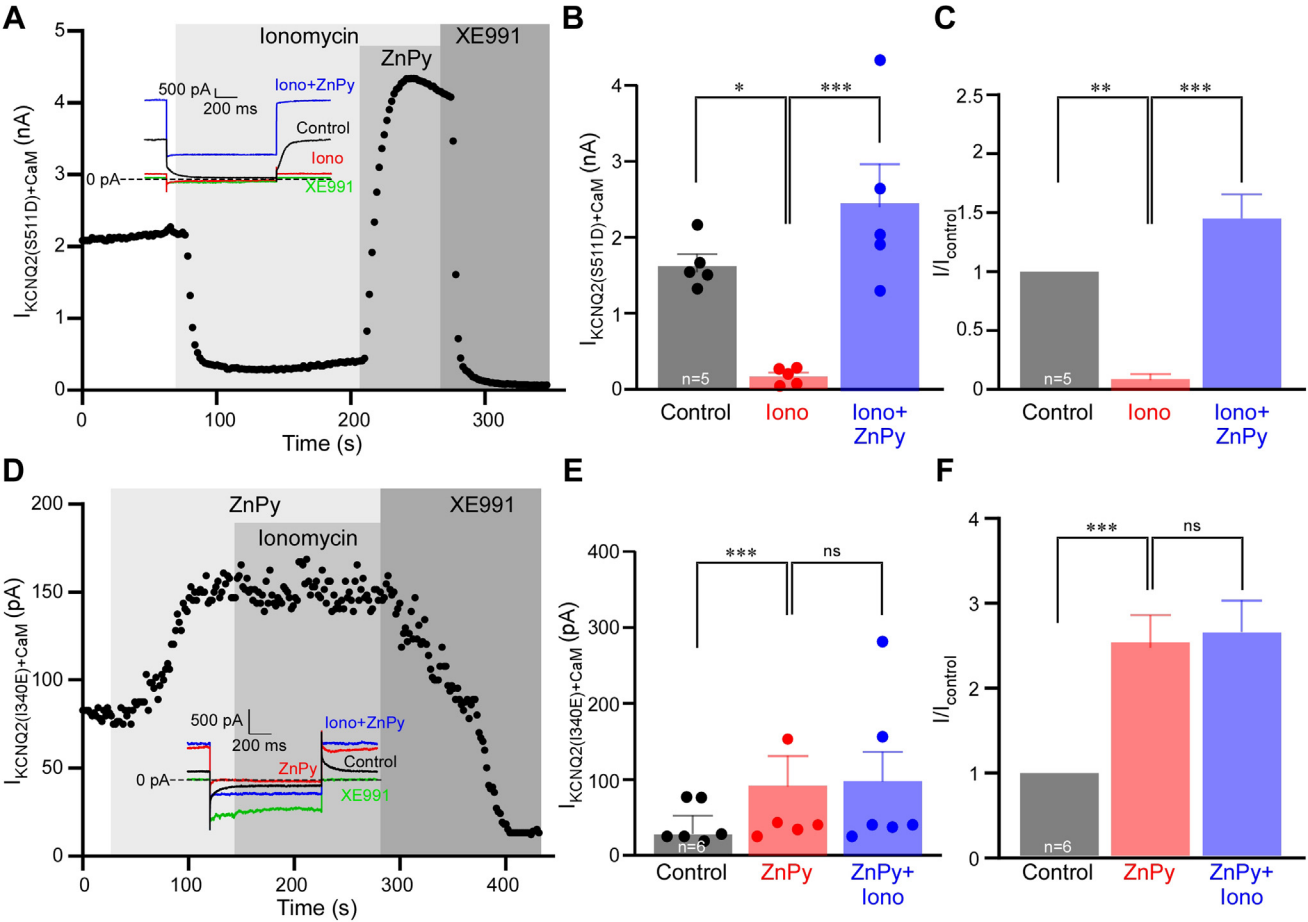
**Action of Ca**^**2+**^**and Zn**^**2+**^**ionophores on Kv7.2 calmodulin (CaM) binding mutants.***A*, Chinese hamster ovary (CHO) cells were cotransfected with Kv7.2 (S511D) and CaM; time course of the effects of ionomycin (5 μM), zinc pyrithione (ZnPy) (10 μM), and XE991 (10 μM) is shown. Recording conditions and labeling is similar to that used for Figure 1*A*. *B* and *C*, summary of experiments shown in *A*, n = 5. *Asterisks* depict a significant difference between the groups indicated by *connector lines*; ∗*p* < 0.05, ∗∗*p* < 0.01, and ∗∗∗*p* < 0.001 (repeated-measures ANOVA with Bonferroni post hoc test). *D*, experiment similar to that shown in *A*, but Kv7.2 (I340E) was overexpressed and ZnPy was applied first, followed by the application of ionomycin (still in the presence of ZnPy). *E* and *F*, summary of experiments shown in *D*, n = 6. *Asterisks* depict a significant difference between the groups indicated by *connector lines*; ∗∗∗*p* < 0.001 (repeated-measures ANOVA with Bonferroni post hoc test).

Next, we coexpressed both mutants with CaM and tested their sensitivity to Ca^2+^ and Zn^2+^ elevations. Kv7.2 (S511D) behaved similarly to the WT Kv7.2 and displayed robust sensitivity to Ca^2+^/ionomycin, which was completely reverted by 10 μM ZnPy (Fig. 5, *A*–*C*). Compared with basal level, Kv7.2 (S511D) current amplitude was increased by 1.46 ± 0.21-fold, which was comparable with the efficacy of ZnPy to augment WT Kv7.2 current amplitude (Fig. 1, *A*–*F*). Current amplitude generated by Kv7.2 (I340E) with CaM was too small for meaningful quantification of inhibitory effect of Ca^2+^/ionomycin; hence, we first applied 10 μM ZnPy, which produced more that twofold augmentation of current amplitude (Fig. 5, *D*–*F*). Then, we added 5 μM ionomycin, still in the presence of ZnPy. As in the case of WT Kv7.2, there was no inhibition, and the current was fully inhibited by XE991. In combination, the experiments presented in Figure 3, Figure 4, Figure 5 suggest that impairing CaM binding to Kv7.2 reduces the number of functional channels at the plasma membrane, but the functional channels that did reach plasma membrane are likely to still bind CaM, as suggested previously (41). With respect to the modulation by Ca^2+^ and Zn^2+^, these mutant channels behave similarly to the WT Kv7.2: Ca^2+^ inhibits them and Zn^2+^ removes this inhibition. Inhibition by Ca^2+^ does not hamper Zn^2+^ efficacy to augment channel activity, whereas pretreatment with Zn^2+^ renders the channel insensitive to inhibition by Ca^2+^.

### Reducing CaM binding to Kv7.3 unmasks sensitivity to zinc

**Figure 6:**
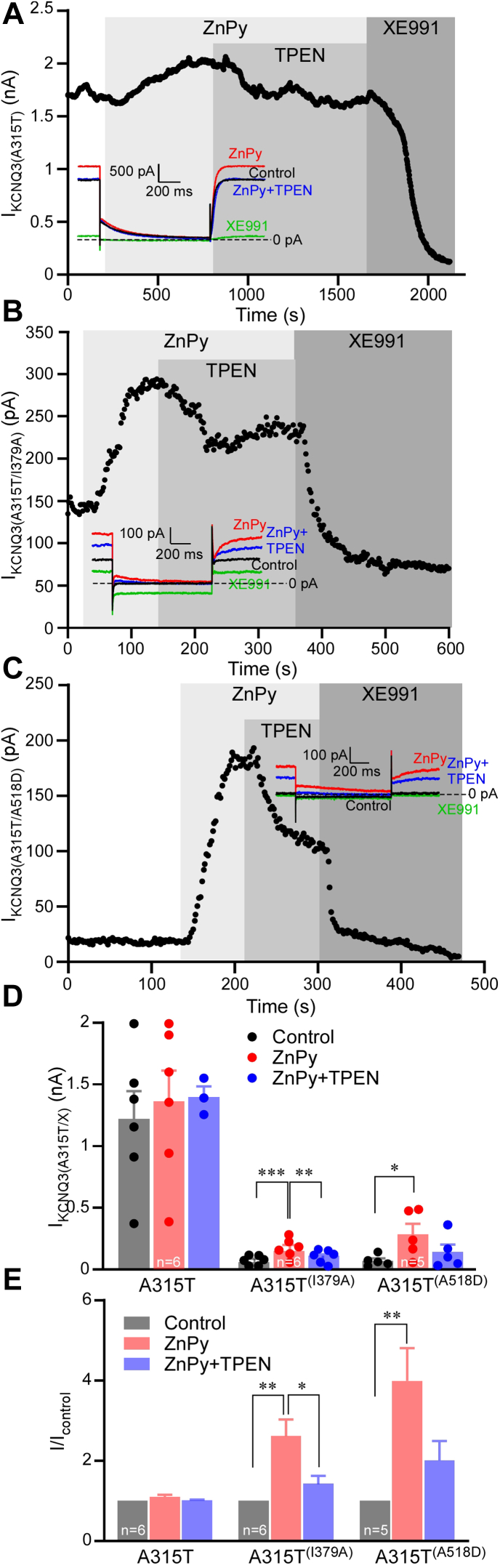
**Reducing calmodulin (CaM) binding to Kv7.3 unmasks sensitivity to zinc.***A*–*C*, Chinese hamster ovary (CHO) cells were transfected with Kv7.3 (A315T) (*A*), Kv7.3 (A315T/I379A) (*B*), or Kv7.3 (A315T/A518D) (*C*); time courses of the effects of zinc pyrithione (ZnPy) (10 μM), *N*,*N*,*N*′,*N*′,tetrakis(2-pyridylmethyl)ethylenediaminepentaethylene (TPEN) (20 μM) and XE991 (10 μM) are shown. Recording conditions and labeling is similar to that used for Figure 1*A*. *D* and *E*, summary of experiments shown in *A*–*C*, numbers of experiments are indicated within the *bars*. *Asterisks* depict a significant difference between the groups indicated by *connector lines*; ∗*p* < 0.05, ∗∗*p* < 0.01, and ∗∗∗*p* < 0.001 (repeated-measures ANOVA with Bonferroni post hoc test).

Kv7.3 was shown to have marginal sensitivity to zinc (20, 21). The likely explanation to that is the intrinsically high tonic open probability (*P*_o_) of Kv7.3, which is close to 1 at saturating voltages (43), owing to uniquely high (amongst the Kv7s) apparent affinity of Kv7.3 to its positive regulator, PIP_2_ (43, 44). Thus, basal PIP_2_ levels in cells are usually sufficient to maintain tonic maximal *P*_o_ of this channel near unity, preventing further augmentation by Zn^2+^ (20). We next tested the relationships between the Kv7.3 current amplitude, CaM binding, and zinc modulation. Because the Kv7.3 expresses poorly as a homomer (17, 45), we used Kv7.3 with a pore domain mutation, A315T, which increases channel activity without changing apparent PIP_2_ affinity (44, 45, 46). As previously reported, Kv7.3 (A315T) was only marginally (and not significantly) affected by 10 μM ZnPy (Fig. 6, *A*, *D* and *E*). We then mutated residues I379 and A518, which in Kv7.3 are at positions equivalent to I340 and S511 in Kv7.2, to impair CaM binding to helix A or B, respectively. We used Kv7.3 A315T/I379A and A315T/A518D mutants that were shown to have reduced CaM binding previously (42). Both double mutants displayed much reduced current amplitude, as compared with Kv7.3 A315T single-mutant channel, but in both cases, 10 μM ZnPy was able to augment current amplitude, and this effect was significantly more robust than in the case of the Kv7.3 A315T single mutant (Fig. 6, *B*–*E*). The most striking was the case of the Kv7.3 A315T/A518D. Consistent with a previous report (42), in CHO cells transfected with this double mutant, there was virtually no measurable current (and, therefore, this mutant was previously considered nonfunctional). Yet, addition of 10 μM ZnPy revealed a clearly M-like slow outward K^+^ current, which was abolished by XE991 (Fig. 6, *C* and *D*). Overall, ZnPy produced 1.08 ± 0.04-fold (n = 5) current augmentation of Kv7.3 A315T single mutant, 2.62 ± 0.4-fold (n = 6; *p* < 0.01) augmentation of Kv7.3 A315T/I379A, and staggering 3.99 ± 0.81-fold (n = 5; *p* < 0.01) augmentation of A315T/A518D. These experiments reveal that CaM binding deficiency impairs Kv7.3 channel function, but this can be rescued by intracellular Zn^2+^ to a significant degree. Even a channel previously characterized as nonfunctional (Kv7.3 A315T/A518D) can be “brought to life” in the presence of Zn^2+^.

Interestingly, at higher concentrations, ionomycin has been shown to activate Ca^2+^-sensitive PLCδ delta and deplete membrane PIP_2_ (26). In this study, 1 and 3 μM ionomycin did not produce PIP_2_ depletion in CHO cells, whereas at 10 μM, significant depletion was seen. Since Kv7 channels are PIP_2_ dependent (14, 15, 43), a potential depletion of PIP_2_ in our ionomycin experiments would significantly complicate our interpretations. Hence, we used optical PIP_2_ reporter, YFP-tubby (R332H mutated) (47, 48), to test if 5 μM ionomycin produces any effect on the plasma membrane PIP_2_ levels (Fig. S3). Similar to 1 μM and 3 μM, reported by Kosenko and Hoshi (26), 5 μM ionomycin did not produce any measurable change in PIP_2_ probe localization, whereas activation of M1 muscarinic receptors (cotransfected together with the YFP-tubby, as a positive control) did induce robust translocation of the probe. Hence, we conclude that under our experimental conditions, 5 μM ionomycin does not produce measurable effect on PIP_2_ abundance, and the inhibitory effect of ionomycin on Kv7 channels is likely to be mediated by Ca^2+^/CaM.

### Effect of zinc on Kv7 channels is not mediated by CaM

**Figure 7:**
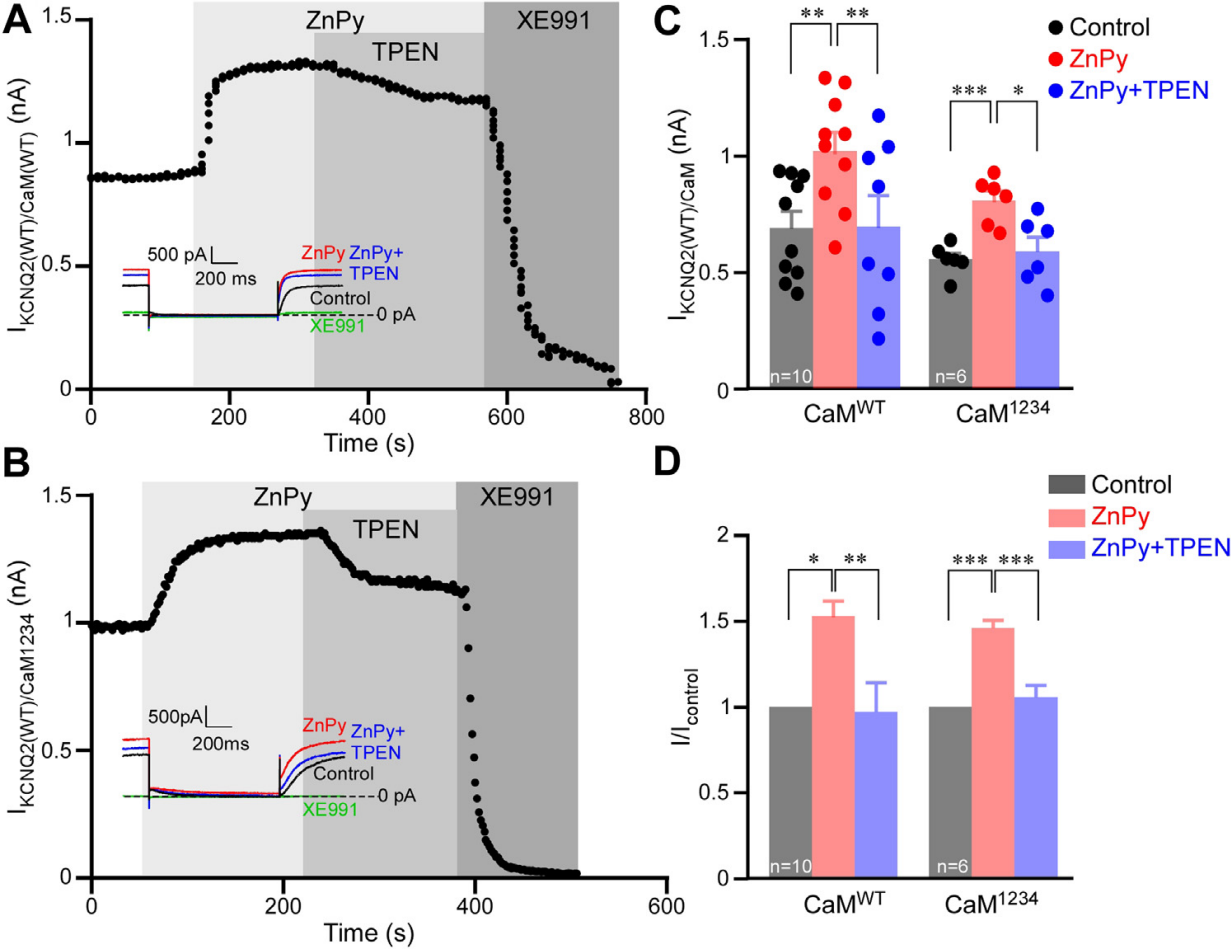
**Modulation of Kv7.2 by zinc is not mediated by calmodulin (CaM).***A* and *B*, Chinese hamster ovary (CHO) cells were transfected with WT Kv7.2 and either WT CaM (*A*) or CaM1234 (*B*); time courses of the effects of zinc pyrithione (ZnPy) (10 μM), *N*,*N*,*N*′,*N*′,tetrakis(2-pyridylmethyl)ethylenediaminepentaethylene (TPEN) (20 μM), and XE991 (10 μM) are shown. Recording conditions and labeling is similar to that used for Figure 1*A*. *C* and *D*, summary of experiments shown in *A* and *B*. Numbers of experiments are indicated within the *bars*. *Asterisks* depict a significant difference between the groups indicated by *connector lines*; ∗*p* < 0.05, ∗∗*p* < 0.01, and ∗∗∗*p* < 0.001 (repeated-measures ANOVA with Bonferroni post hoc test).

Because CaM was shown to be able to bind not only Ca^2+^ but also Zn^2+^ (albeit with rather low upper-micromolar affinity (49, 50)) and because of the fact that zinc-binding site of Kv7 channels remains elusive (20), we tested if Kv7 channel modulation by zinc is actually mediated by CaM. We tested if Kv7.2 is still sensitive to ZnPy when assembled with CaM in which all four EF hands are mutated and locked in the apo state (CaM1234 (51, 52)). As shown in Figure 7, Kv7.2 coexpressed with either WT CaM (Fig. 7, *A* and *C*, D) or with CaM1234 (Fig. 7, *B*–*D*) displayed very similar response to 10 μM ZnPy. Thus, it is unlikely that the augmenting effect of Zn^2+^ on Kv7 channels is mediated by CaM.

## Discussion

In this study, we investigated relationships between Kv7 channel inhibition by Ca^2+^/CaM and the potentiating effect of zinc. Our main findings are as follows: (i) Zn^2+^ ionophore ZnPy can completely prevent inhibition of Kv7.2 by Ca^2+^/CaM. Likewise, ZnPy can reverse Ca^2+^/CaM-induced inhibition of Kv7.2. In either case, the presence of Ca^2+^ does not significantly affect the efficacy of the augmentation of current amplitude by ZnPy. Consistent with a previous report (20), augmenting effect of ZnPy is reversed by Zn^2+^ chelator, TPEN (still in the presence of pyrithione), indicating that Zn^2+^ is the active moiety mediating the effect of ZnPy. (ii) In the presence of both Ca^2+^ (delivered by ionomycin) and Zn^2+^ (delivered by ZnPy), Kv7.2 channels lose most of their voltage dependence and lock in the open state. (iii) Consistent with the previous literature (41, 42), mutations that interfere with CaM binding to Kv7.2 and Kv7.3 reduce channel membrane abundance and activity, but these mutants retain sensitivity to zinc. Moreover, its relative efficacy on mutants is generally greater. (iv) Zinc sensitivity of Kv7 channels is unlikely to be mediated by CaM.

Altogether, these results suggest that intracellular zinc is a remarkable stabilizer of Kv7 channel activity. In addition to previously reported ability of zinc to remove Kv7 channel dependence on PIP_2_ (20), we now show that it renders Kv7 channels insensitive to Ca^2+^-mediated inhibition. More than that, even structurally handicapped channels with impaired ability to bind CaM can be “resurrected” by zinc. A striking example of that is Kv7.3 A315T/A518D mutant: it displays virtually no activity under basal conditions and was reported as “nonfunctional” before (42), but it can be significantly activated in the presence of zinc (Fig. 6).

Functional relationships between Kv7 channel activity, CaM and Ca^2+^ are complex. It is presently accepted that all Kv7 channels require CaM for functional assembly at the plasma membrane as mutations that impair CaM binding usually negatively impact both, membrane abundance and activity of Kv7 channels (19, 29, 30, 42, 53). CaM also mediates Ca^2+^-dependent modulation on Kv7 channels (17, 18, 24, 25). The effect of Ca^2+^ elevation on Kv7–CaM complex is generally inhibitory (17, 18, 24, 26, 33, 54) and was suggested to contribute to Kv7 inhibition by Gq-coupled GPCR (see Ref. (9) for review). However, Kv7.1 seems to be different in this regard, as Ca^2+^ activates it, instead of inhibiting (28, 32, 34). The mechanism(s) of CaM/Ca^2+^ modulation of Kv7 channels are unclear, but most hypotheses consider convergence of CaM, Ca^2+^, and PIP_2_, whereby conformational changes induced by Ca^2+^ binding to CaM modify channel interactions with PIP_2_. Indeed, PIP_2_ interacts with several channel regions, which are also involved in the interaction with CaM, including the C-terminal HA–HB region (32, 55, 56, 57, 58), S2–S3 loop, and S4–S5 loop (39, 55, 56, 57, 59). Hence, if we are to hypothesize that Ca^2+^/CaM inhibits Kv7 channel activity by disrupting its interaction with PIP_2_, then, the facts that intracellular Zn^2+^ prevents/reverts Ca^2+^/CaM-mediated inhibition of Kv7s reported here, and our earlier observation that Zn^2+^ removes Kv7 channel’s requirement for PIP_2_ (20), may have a common underlying mechanism. For instance, if Zn^2+^ stabilizes open state of the channel in the absence of PIP_2_, then any channel modulation or modification that acts by decreasing channel PIP_2_ affinity (or otherwise increasing PIP_2_ dependence) may fail to manifest.

An additional possibility to be considered here is that HA/HB mutations that reduce CaM binding to Kv7 channels may also disturb C-terminal PIP_2_-binding sites, specifically, the ones in the HA–HB linker (55, 58) and at cytosolic end of the S6 (15, 36, 55, 59). Hence, small or even negligible (as in the case of Kv7.3 A315T/A518D) currents recorded from these mutants may reflect their reduced PIP_2_ affinity. In this case, the action of Zn^2+^ would be again in stabilizing channel opening, independent of PIP_2_. Future structural insights will be required to test these speculations.

Regardless of the aforementioned intricacies, the fact that intracellular Zn^2+^ abolishes both, the PIP_2_-mediated (20) and Ca^2+^/CaM-mediated (present study) Kv7 channel inhibition, makes zinc-bound Kv7 channels resistant to most physiological inhibitory modulation, for example, by acetylcholine, glutamate, bradykinin, histamine, angiotensin II, and so on. In support of this speculation, inhibition of Kv7.2, Kv7.3 (23) and Kv7.4 (20) by M1 muscarinic acetylcholine receptors was virtually abolished by zinc. This may have significant implication for neurotransmission in the brain. Zinc is highly concentrated within synaptic vesicles in a subset of glutamatergic neurons in hippocampus and some other brain regions, such as olfactory bulb (reviewed in Ref. (60)); it can also be found in some other neuronal subpopulations, such as spinal GABAergic neurons (61). Synaptic release of neurotransmitters from these “high-zinc” neurons results in free Zn^2+^ release to synaptic cleft, where it can reach over 100 μM levels (60). Significant amounts of Zn^2+^ can enter both postsynaptic and presynaptic terminals *via* AMPA receptors (3, 4, 5) as well as through some other cationic channels. Neurotransmitters, acting *via* G_q_-coupled metabotropic receptors (*e.g.*, glutamate *via* group I mGluRs (62)), could inhibit M channels causing further excitation. Additional negative impact on M channels could arise from activity-dependent Ca^2+^ accumulation. Thus, synaptically released zinc may be utilized to protect M channels from these inhibitory actions, preventing overexcitability. Therefore, zinc-mediated Kv7 channel stabilization may have an important role in the maintenance of appropriate firing rates in neuronal circuits.

## Experimental procedures

### Cell culture and transfection

CHO cells were obtained from Kunming Institute of Zoology, Chinese Academy of Sciences. Cells were grown in T25 flasks in Dulbecco's modified Eagle's medium (DMEM)/F12 medium with 10% fetal bovine serum and 0.1% penicillin/streptomycin in a humidified incubator at 37 °C (5% CO_2_) and passaged about every 2 days. Rat dorsal root ganglion neurons were isolated and cultured as described before (63). All animal experiments were performed in accordance with the Animal Care and Ethical Committee of Hebei Medical University (approval number: IACUC-Hebmu-2020007). CHO cells were transfected with human *KCNQ2* (Y15065) or *KCNQ3* (NM_004519) cDNA subcloned into pCDNA3.1 (Youbio), with or without WT CaM (NP_008819.1) or CaM1234 (a gift from Dr Zhiqiang Yan, Fudan University). Point mutations in *KCNQ2* and *KCNQ3* were produced by Youbio. YFP-tubby (tubby-R332H-cYFP was a gift from Andrew Tinker (UCL)). Human M1 receptor cDNA was a gift from Hailin Zhang (Hebei Medical University). For transfection, cells were cultured on 24-well plates and transfected with FuGENE HD Transfection Reagent (Promega), according to the manufacturer’s instructions. Twenty-four hours later, the cells were plated onto poly-l-lysine–coated coverslip chips; experiments were performed 48 to 96 h after transfection. As a marker for successfully transfected cells, cDNA-encoding GFP was cotransfected together with the other genes of interest.

### Patch-clamp recording

The perforated patch configuration of the patch-clamp technique was used to voltage clamp and dialyze cells. Recordings were performed at room temperature (22–25 °C) using amphotericin B (0.5 mg/ml) as a pore-forming agent. Pipettes were pulled from borosilicate glass capillaries using a Flaming/Brown micropipette puller P-97 and had resistances of 2 to 5 MΩ. Currents were amplified by the EPC10 USB amplifier (HEKA) and recorded using the Patchmaster software (v2x90.4, October 30, 2018; HEKA). Capacitance current artifacts were cancelled, and series resistance was compensated by ∼80%. To evaluate the amplitude of Kv7 currents, CHO cells were held at 0 mV, and 1000 ms hyperpolarizing steps to –60 mV, followed by a 500 ms pulse back to 0 mV, were applied every 3 s. In dorsal root ganglion neuron recordings, similar voltage protocol was used, but cells were held at −30 mV. To investigate current–voltage relationships, CHO cells were held at −80 mV, and the currents were elicited by a series of 1500 ms steps from −70 to +50 mV in 10 mV increments. The G–V curves were fit by the Boltzmann equation:

$$G=G_{\min}+\left(G_{\max}{-G}_{\min}\right)/\left(1+e^{\left(V_{0}-V\right)/\text{dx}}\right)\text{,}$$

where *G*_max_ is the maximum conductance and *G*_min_ is the minimum conductance.

### Western blot

Human embryonic kidney 293T cells (Kunming Institute of Zoology, Chinese Academy of Sciences) were grown in 100 mm cell culture dishes in DMEM containing 10% fetal bovine serum and 1% penicillin/streptomycin (complete medium) at 37 C in a 5% CO_2_ incubator. Cells were transfected with FLAG-tagged WT *KCNQ2* and *KCNQ2* (I340E) or with HA-tagged *KCNQ2* (S511D) by using polyethyleneimine transfection reagent (Polysciences), at plasmid:polyethyleneimine ratio of 1:3. In experiments with ZnPy application, 10 μM ZnPy or vehicle, cells were applied for 2 min before harvest. Membrane proteins were extracted and separated from nonmembrane proteins by Minute Plasma Membrane Protein Isolation and Cell Fractionation Kit (Invent). Membrane protein fraction was collected and dissolved in Minute Nondenatured Protein Solubilization Reagent (Invent). Protein concentration was measured with Pierce BCA Protein Assay Kit (Solarbio). Samples were denatured at 95 °C for 5 min and separated on a gradient SDS-PAGE gel. Samples were transferred to a 0.2 μm polyvinylidene difluoride membrane (Millipore) at 120 V for 90 min on ice and blocked with 5% skim milk powder in Tris-buffered saline with Tween-20 on a rocking platform for 1 h at room temperature. The membranes were then incubated with following primary antibodies overnight at 4 °C: anti-DDDDK-tag monoclonal antibody (catalog no.: M185-3L; MBL; 0.1 μg/ml); antihemagglutinin-tag monoclonal antibody (catalog no.: M180-3; MBL; 0.1 μg/ml); anti-β-actin monoclonal antibody (catalog no.: AC026; ABclonal; 1:5000 dilution); and anti-CaM monoclonal antibody (catalog no.: 05-173; Merck; 1:2000 dilution). Next, membranes were incubated with respective secondary antibody in 5% skim milk powder: antimouse (goat) DyLight TM 800 (catalog no.: 610-145-002, Rockland; 1:10,000 dilution) or anti-rabbit (goat) DyLight TM 800 (catalog no.: 611-145-002, Rockland; 1:10,000 dilution). Protein signals were detected using Odyssey infrared fluorescence scanning imaging system. The intensity of each protein band was analyzed using ImageJ (National Institute of Health).

### Co-IP

Cells were prepared and transfected as described in previous section. Cells were lysed using cell lysis buffer for Western blot and IP (Beyotime). Protein concentration was measured with Pierce BCA Protein Assay Kit. The total protein from each sample was precleaned and divided into three groups: input/immunoglobulin G (IgG)/co-IP. The “input” sample was mixed with 5× loading buffer at a ratio of 4:1, heated at 95 °C for 5 min, and stored at −20 °C until further analysis. The “co-IP” samples were incubated with anti-FLAG antibody (catalog no.: M185-3L; MBL; 2 μg) or antihemagglutinin-tag monoclonal antibody (catalog no.: M180-3; MBL; 2 μg) at 4 °C overnight. The “IgG” samples were incubated with mouse IgG (Santa Cruz) under the same conditions. Protein A/G PLUS-Agarose beads (50 μl; Santa Cruz) were added to the “co-IP” and “IgG” samples and incubated at 4 °C for 4 h with rotation. These beads were washed five times with PBS buffer and centrifuged at 12,000*g* at 4 °C for 5 min. The pellet was resuspended with 60 μl of loading buffer, boiled for 2 to 3 min, and then subjected to Western blot analysis.

### Solutions and reagents

The external solution for patch-clamp recording from the CHO cells contained (in millimolar): 160 NaCl, 2.5 KCl, 2 CaCl_2_, 1 MgCl_2_, and 10 Hepes (pH adjusted to 7.4 with NaOH). The pipette solution for perforated patch contained (in millimolar): 90 potassium acetate, 20 KCl, 1 CaCl_2_, 3 MgCl_2_, 40 Hepes, 3 EGTA, and 0.5 mg/ml amphotericin B (pH adjusted to 7.4 with KOH). In whole-cell experiments with “low” (50 nM) and “high” (1 μM) [Ca^2+^]_I_, the pipette solutions were (in millimolar): 130 KCl, 5 MgCl_2_, 5 EGTA, 3 K-ATP, and 5 Hepes. To obtain 50 nm and 1 μM cytosolic-free [Ca^2+^], 1.94 or 4.63 mM CaCl_2_ was added (as calculated with MAXCHELATOR; https://somapp.ucdmc.ucdavis.edu/pharmacology/bers/maxchelator/). Reagents were obtained as follows: ZnPy, TPEN, XE991, amphotericin B (Sigma–Aldrich); ionomycin (Cayman Chemical); DMEM-F12, DMEM, and fetal bovine serum (Gibco).

### Statistical analysis

The data were analyzed and plotted with Origin 9.1 (OriginLab) and GraphPad Prism 8 (GraphPad Software). Data are presented as means ± SEM and were statistically compared using Student’s paired or unpaired *t* test, one-way ANOVA, or repeated-measures ANOVA with Tukey’s or Bonferroni’s post hoc tests as appropriate.

## Data availability

All data are available in the main text and presented as scatter plots.

## Supporting information

This article contains supporting information.

## Conflict of interest

The authors declare that they have no conflicts of interest with the contents of this article.
