# Supplementary material for: Intracellular zinc protects Kv7 K+ channels from Ca2+/calmodulin-mediated inhibition
Source: J Biol Chem. 2022 Dec 20;299(2):102819. doi: 10.1016/j.jbc.2022.102819 (PMC9852549; doi:10.1016/j.jbc.2022.102819)
Supplement: Supplemental Figures S1–S3 [file mmc1.pdf]

Intracellular zinc protects Kv7 K^+ channels from Ca^{2+}/calmodulin-mediated inhibition

Xinhe Yang, Shuai Chen, Shuo Zhang, Sai Shi, Rui Zong, Yiting Gao, Bingcai Guan, Nikita Gamper, Haixia Gao

Supporting information

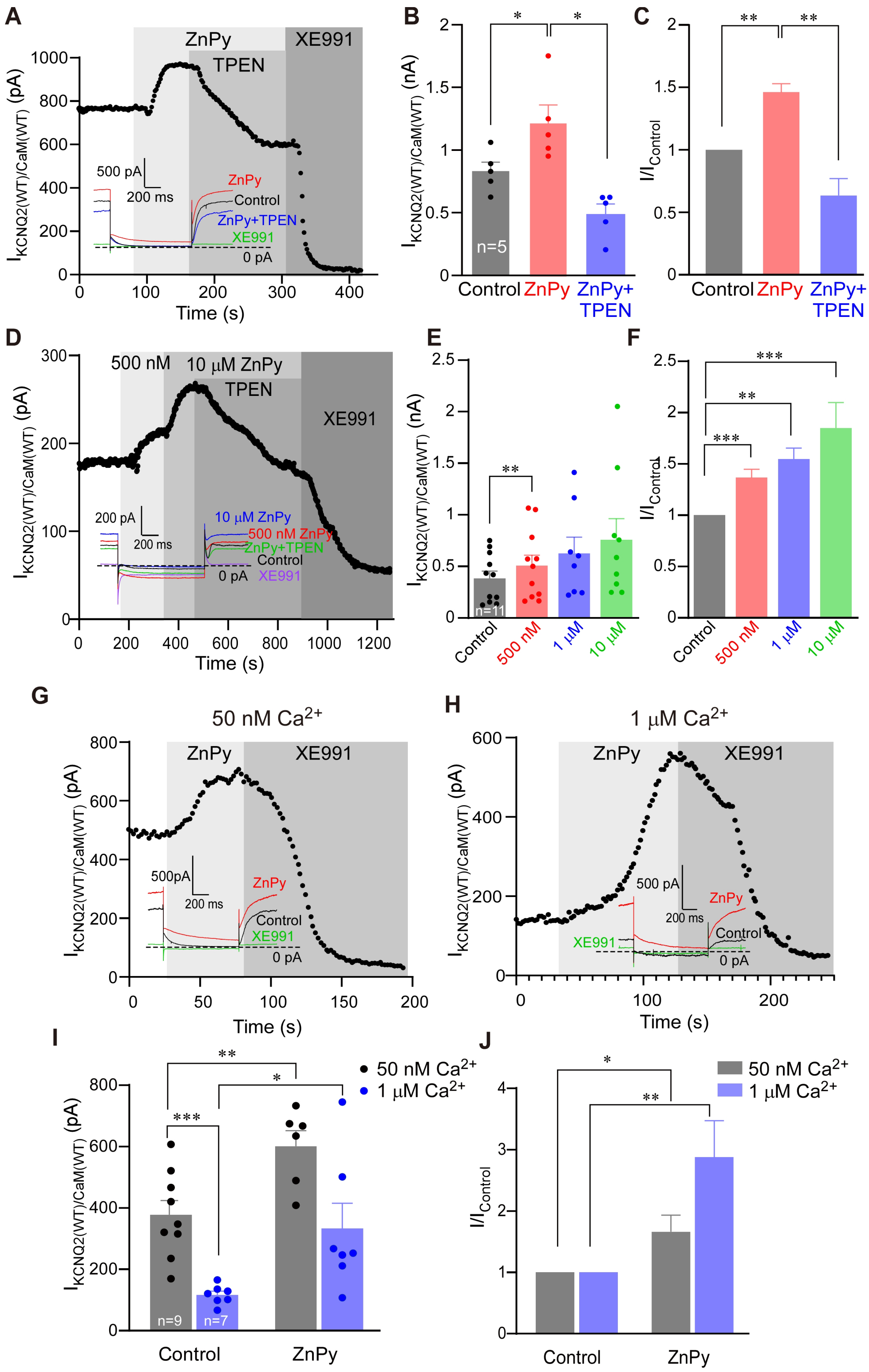

Supplemental Figure 1

Supplemental Figure 1. Additional experiments on modulation of Kv7.2 currents by zinc and calcium. A, Perforated patch-clamp recording from CHO cell co-transfected with Kv7.2 and CaM, showing the time course for the effects of zinc ionophore, ZnPy (10 μM) and zinc chelator, TPEN (30 μM), as labelled. At the end of the recording a specific Kv7 channel inhibitor, XE991 (10 μM), was applied. Vertical grey bars indicate periods of drug application. **B, C,** Summary of the experiments shown in A. Mean current amplitudes are summarised in B and normalised current amplitudes (relative to basal amplitude, $I_{control}$) are summarised in C; n=5. Asterisks depict a significant difference between the groups indicated by connector lines; *p<0.05, **p<0.01 (repeated measures ANOVA with Bonferroni post-hoc test). **D,** CHO cells were co-transfected with Kv7.2 and CaM; time course of the effects of ZnPy (500 nM and 10 μM), TPEN (20 μM) and XE991 (10 μM) is shown. Recording conditions and labelling are similar to that used in panel A. **E, F,** Summary of experiments shown in D, n=11. Asterisks depict a significant difference between the groups indicated by connector lines; **p<0.01, ***p<0.001 (repeated measures ANOVA with Bonferroni post-hoc test). **G, H,** Whole-cell recordings of the effects of ZnPy (10 μM) on Kv7.2/CaM currents in CHO cells with intracellular [Ca^{2+}] clamped to 50 nM (G) or 1 μM (H). **I, J**, Summary of experiments shown in G (n=9) and H (n=7). Asterisks depict a significant difference between the groups indicated by connector lines; *p<0.05, **p<0.01 (repeated measures ANOVA with Bonferroni post-hoc test).

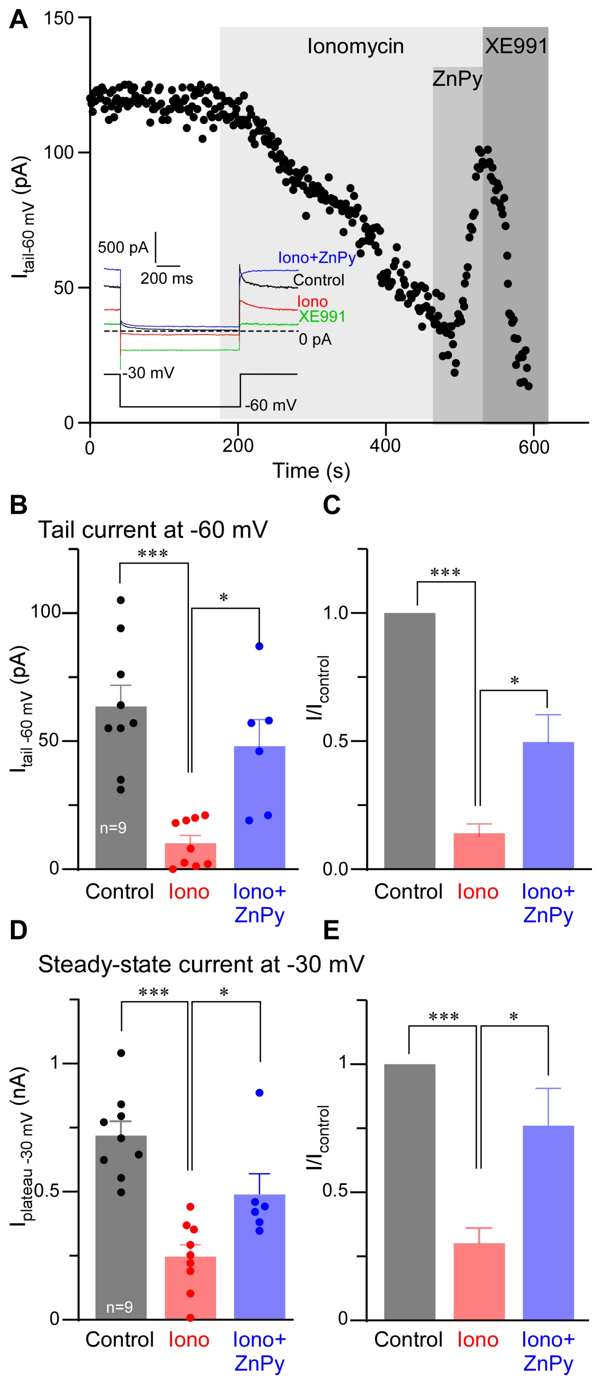

Supplemental Figure 2

Supplemental Figure 2. Modulation of M-like current in cultured DRG neurons by zinc and calcium. A, Perforated patch-clamp recording from a cultured small-diameter DRG neuron, showing the time course for the effects of ionomycin (5 µM), ZnPy (10 µM) and XE991 (10 µM). Representative current traces and voltage protocol are shown in the inset. **B-E,** Summary of the experiments shown in A. Mean tail current amplitudes at -60 mV are summarised in B (n=9), mean steady-state current amplitudes at -30 mV are summarised in D (n=9). Normalised current amplitudes for tail currents (and steady-state currents are summarised in C and E, respectively. Asterisks depict a significant difference between the groups indicated by connector lines; *p<0.05, ***p<0.001 (repeated measures ANOVA with Bonferroni post-hoc test).

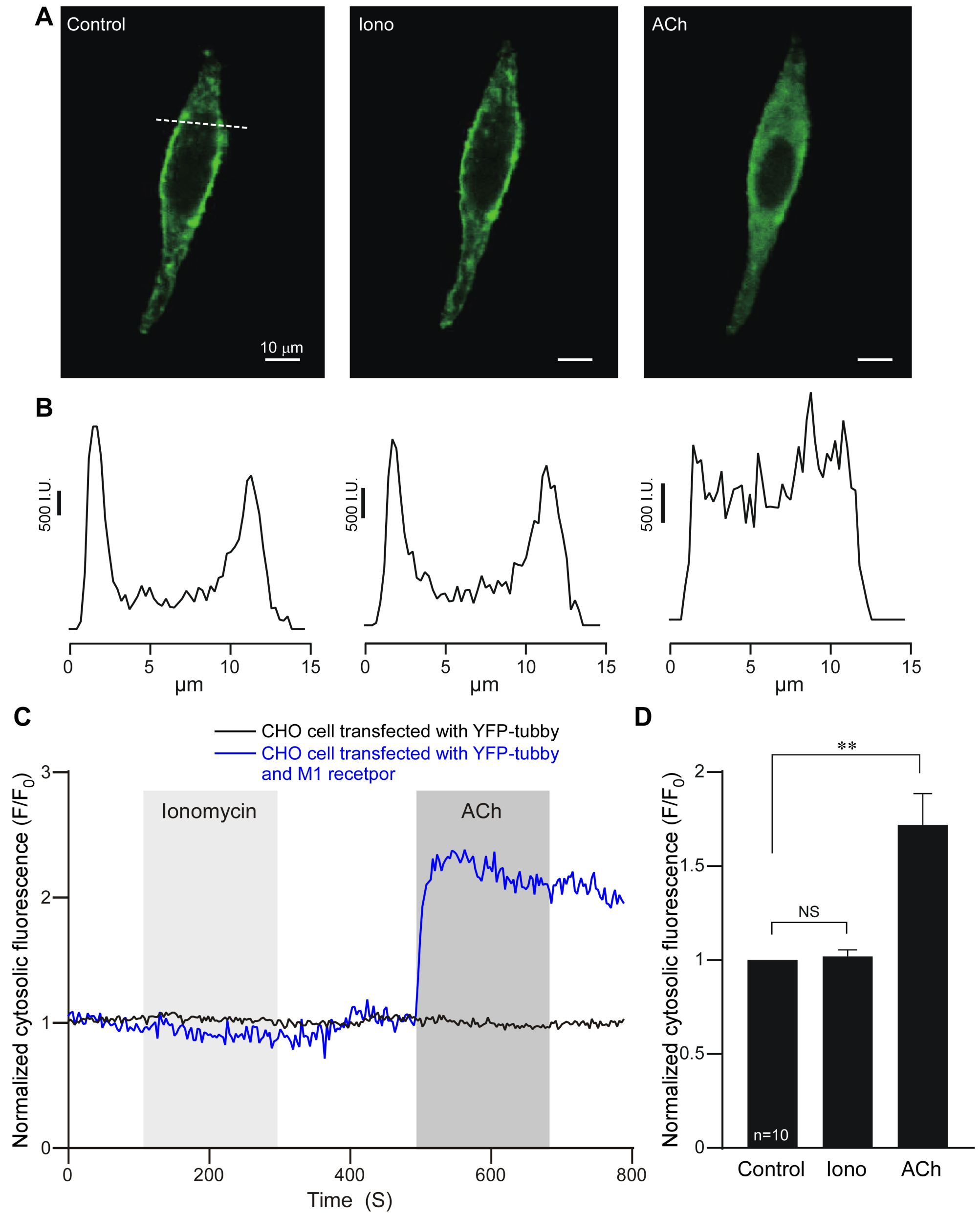

Supplemental Figure 3

Supplemental Figure 3. Ionomycin at 5 μM does not induce significant plasma membrane PIP_2 depletion in CHO cells. CHO cells were transfected with YFP-tubby PIP_2 probe and M1 muscarinic receptor. **A,** confocal micrographs of the transfected CHO cell before (Control), during application of 5 μM ionomycin (Iono) and during subsequent application of 500 nM acetylcholine (Ach). **B,** panels show the corresponding line intensity plots across the areas indicated by the dotted line in the left image in panel A. Scale bars show absolute pixel intensity ($\times 10^2$). **C,** normalised increase in cytosolic fluorescence (F/F_0) in example CHO cells transfected either with YFP-tubby only (black line) or with YFP-tubby and M1 receptor (blue line). **D,** Summary of experiments shown in C (n=10). Asterisks depict a significant difference between the groups indicated by connector lines; $^{**}p<0.01$; NS depicts no significant difference (repeated measures ANOVA with Bonferroni post-hoc test).
